# Local floral resources and edge density within the urban ecosystem promote larger and less variable body size in the great banded furrow bee, *Halictus scabiosae*

**DOI:** 10.1186/s12862-025-02416-5

**Published:** 2025-07-31

**Authors:** Lucie M. Baltz, Julienne de Vastey, Hanna Gardein, Felix Klaus, Henri Greil, Robert J. Paxton, Panagiotis Theodorou

**Affiliations:** 1 General Zoology, Institute of Biology, Martin Luther University Halle-Wittenberg, Hoher Weg 8, Halle (Saale), 06120 Germany; 2 Washington & Lee University, 204 W. Washington St, Lexington, VA 24450 USA; 3 Institute for Bee Protection, Julius Kühn Institute (JKI)– Federal Research Centre for Cultivated Plants, Messeweg 11/12, Braunschweig, 38104 Germany; 4 German Centre for Integrative Biodiversity Research (iDiv) Halle-Jena-Leipzig, Puschstrasse 4, 04103 Leipzig, Germany

**Keywords:** Cities, Host plants, Intertegular distance, Urbanisation, Food resources, Semi-natural cover, Edge density

## Abstract

An organism’s body size is a fundamental trait linked to its metabolism, life-history and dispersal. In holometabolous insects, whose size is fixed at adult eclosion, body size can be influenced by environmental factors during development (e.g. nutrition and temperature), or by ecological filtering during adulthood. In bees, larger body size has been linked to advantages in foraging efficiency, thermoregulation, and survival, while excessive variation in body size within populations may indicate developmental instability. Shifts in adult body size have been associated with temperature changes, food resource availability and habitat fragmentation, all of which can be modulated by urbanisation. However, the relationship between urban landscapes and wild bee body size remains poorly understood. In this study we investigated how local floral (food) resources, landscape structure and temperature influence the body size of the great banded furrow bee, *Halictus scabiosae*. Our findings highlight that food resources, semi-natural cover and edge density are the most significant environmental factors influencing body size shifts. Specifically, *H. scabiosae* body size increased with the species richness of flowering host plants at the local patch level. Within sampling sites, body size variation was positively associated with semi-natural cover, suggesting that habitat structure or competition may contribute to size heterogeneity, potentially disrupting size uniformity. Conversely, it was negatively associated with edge density, indicating that a higher amount of ecotones may promote greater size uniformity within populations. Our findings reinforce the idea that enhancing floral resources and improving habitat connectivity through green corridors can support wild bee populations in urban areas.

**Supplementary Information:**

The online version contains supplementary material available at 10.1186/s12862-025-02416-5.

## Background

Urbanisation is a global phenomenon characterised by the transformation of natural areas and arable land into cityscapes designed to meet human needs, often with negative consequences for biodiversity [1–4]. Urban development is accelerating globally, with urban land cover projected to increase by at least 78% by 2050 [5]. This global urban expansion introduces numerous challenges for living organisms, including increased anthropogenic pollution, the proliferation of impervious surfaces, habitat fragmentation and the loss of semi-natural habitats [6, 7]. Additionally, it contributes to rising local temperatures, a phenomenon known as the urban heat island effect [8]. Collectively, these changes pose significant threats to organisms that are unable to adapt or adjust to these novel and challenging environmental conditions [2].

Nevertheless, urban green spaces can provide suitable habitats for many native species [9, 10], including key ecosystem service providers such as wild bees [3]. The abundance of wild bees is primarily influenced by the availability of suitable nesting and flower (food) resources within their flight range [11]. Therefore, it is not surprising that some wild bee species benefit from the novel nesting opportunities and the high availability and seasonal continuity of flower resources in urban green spaces, including private and public gardens, allotments, parks and cemeteries [4, 12–14]. In fact, previous studies have shown that flower-rich urban sites can support higher abundance and species richness of wild bees compared to agriculture areas [15, 16], rural areas [3] and even nature reserves [15, 17].

However, not all wild bee species benefit from urban areas [4, 18, 19]. Anthropogenic changes, including urbanisation, act as ecological filters, selecting for wild bee species based on their functional traits and ecological requirements, thereby shaping species composition [20, 21]. For example, while cavity-nesting bees may benefit from urban structures such as fences and walls, ground-nesting bees often decline as a result of increased impervious surfaces [4, 22]. Indeed, studies have shown that cavity-nesting bees [14, 22–25], social bees [14, 19, 23, 24, 26] and polylectic bee species [24, 27] tend to benefit from urbanisation. Conversely, urban environments appear to negatively impact the abundance of below-ground, small-bodied bees, early spring-flying species and solitary bees [4, 19, 26, 28, 29].

Body size is one of the most important life-history traits as it is closely linked to fitness [30], including fecundity [31], metabolism, dispersal ability and thermoregulation [32]. In bees, body size is highly correlated with foraging range [33], and the amount of pollen and nectar that an individual can carry [34], which may enhance foraging efficiency and pollination success. Larger bees generally outperform smaller bees in these aspects, highlighting the advantages of increased body size [33–35]. As a continuous trait, body size varies not only between species but also within species [36], as it responds to changing abiotic and biotic conditions through adaptation and developmental plasticity [30, 37]. Intraspecific variation in body size among wild bees arises primarily due to differences in temperature and food availability during development [35]. Higher temperatures during the larval stage can accelerate development rates, leading to smaller adult body sizes [38]. Conversely, a greater quality and quantity of food resources during the larval stage can result in larger adult bees [39, 40]. Since body size is an important biological trait shaped by environmental factors and physiological constraints, wild bee species are expected to exhibit intraspecific shifts in body size in response to anthropogenic environmental changes, such as climate change and rising temperatures [41], landscape simplification [42] and urbanisation [43]. Furthermore, as individuals within a population tend to be more similar in size under optimal and stable environmental conditions, high variation in body size may indicate variable and stressful conditions, such as elevated temperatures and patchily distributed food resources, which can be the case in urban areas [44, 45].

However, despite a growing body of research, the effects of urbanisation on intraspecific body size in bees remain inconclusive and difficult to predict. Habitat loss and fragmentation caused by impervious surfaces and the urban heat island effect can have contrasting impacts on body size. For instance, Theodorou et al. [46] found that bumble bee body size in cities increases with road density, a proxy of urban fragmentation. Similarly, Brasil et al. [47] found that *Agapostemon virescens* sweat bees were larger in highly urbanised areas. These studies suggest that cities may act as environmental filters favouring large-bodied individuals within a species. In contrast, Eggenberger et al. [48] found that urban bumble bee workers were smaller than their rural counterparts, supporting the hypothesis that higher temperatures and reduced food resources in cities may lead to smaller individuals within a species. Additionally, other studies have reported negative effects [48–51] or no significant effects of urbanisation on bee body size [50, 52].

The inconsistence findings across studies suggests that intraspecific body size responses are not only species-specific but also context- and site-dependent. Furthermore, most research has compared urban ecosystems with rural or agricultural ecosystems, making it difficult to pinpoint the specific urban environmental factors driving body size shifts in bees. To better understand which environmental features within cities influence body size and its variation, studies conducted within the heterogeneous urban landscape are needed. Such research will help identify how bee body size responds to both local and landscape-level urban factors. Understanding these patterns is crucial for conservation efforts, as body size is a pivotal ecological and evolutionary trait with pervasive effects on individual fitness.

In this study, we extend the research in this field by exploring the relative effects of local floral (food) availability, land surface temperature and landscape composition and configuration on the body size and body size variation of the primitively social wild bee species *Halictus scabiosae*. We hypothesised that these environmental factors would be associated with shifts in *H. scabiosae* body size: specifically, that higher temperatures would correlate with a decrease in body size, while increased floral (food) resources would be associated with an increase in body size. Additionally, we hypothesised that landscape composition and configuration would directly influence body size and body size variation by affecting resource distribution and availability at the landscape level and indirectly by shaping temperature and local floral resource availability.

## Methods

### Study species

*Halictus scabiosae* (Rossi, 1790), commonly known as the great banded furrow bee, is a medium sized (12–14 mm in length), polylectic and socially polymorphic wild bee species belonging to the family Halictidae (sweat bees). Mated females emerge in spring and form nests, either alone or with other females, to raise their offspring. Within these nests, some offspring develop into workers, tasked with foraging and brood care, while others become reproductives, which mate and overwinter to start new nests the following year [53, 54]. After the first brood emerges, the founding female typically remains inside the nest, taking on intranidal tasks, rather than participating in foraging [55, 56]. *Halictus scabiosae* is widely distributed across the western Palearctic and is common throughout most of Europe [57]. It is a ground-nesting bee that prefers xerothermic habitats and, in recent decades, its range has expanded northward, likely due to climate warming [58, 59]. Importantly for our study, *H. scabiosae* is very common in anthropogenic habitats, including cities [54, 60].

### Study area and sampling

**Fig. 1:**
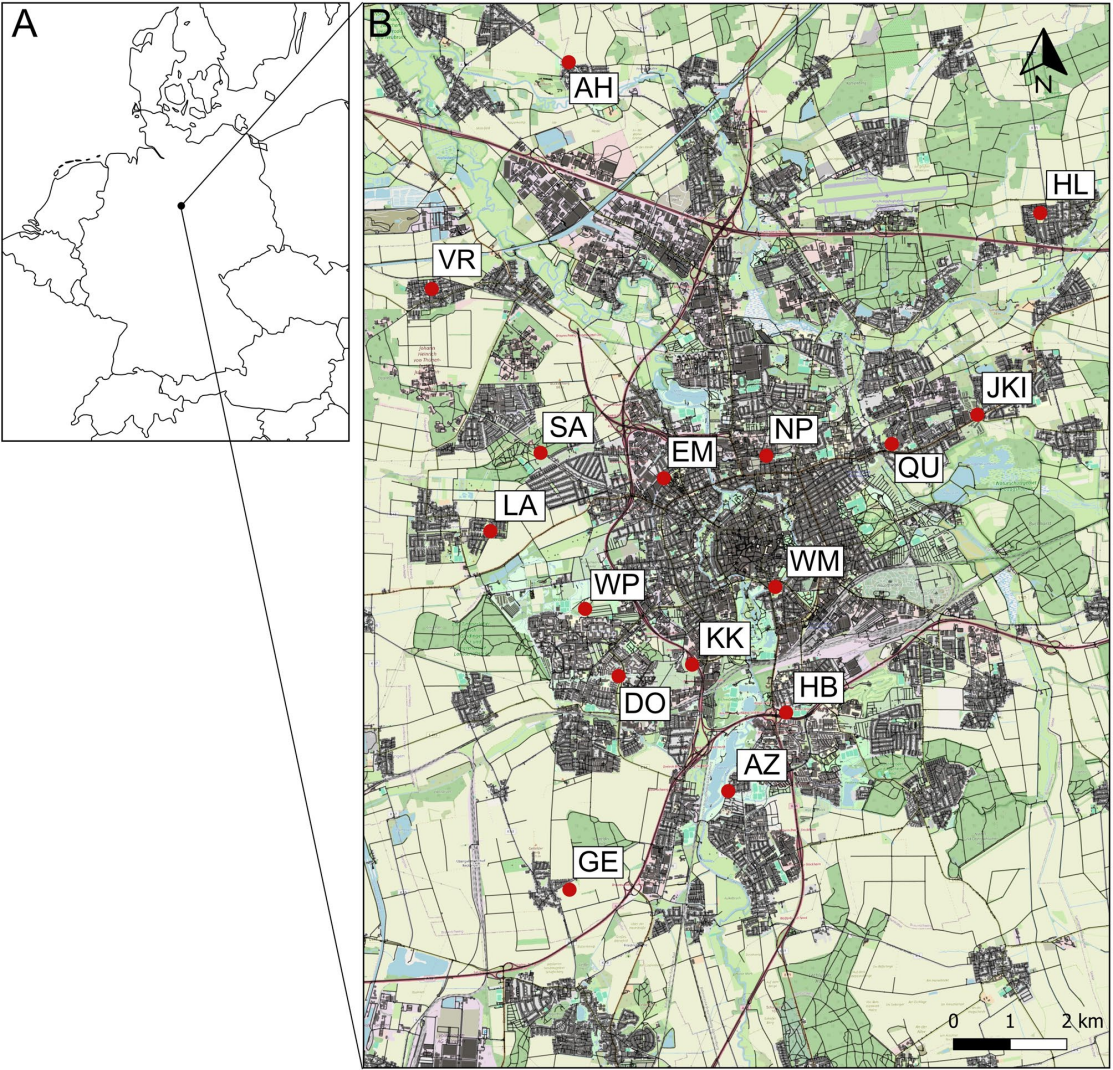
Map of **A** Germany, where the black dot marks the city of Braunschweig, and **B** where the red dots mark the 16 sampling sites in the city of Braunschweig. The ochroid shading represents agricultural areas, the green shading represents semi-natural, forest, and urban green land uses, the grey shadings represent residential area and the black lines represent roads and railway tracks. The map was created with free vector map data licenced by OpenStreetMap Foundation (OSMF) under ODbL (https://opendatacommons.org/licenses/odbl/)

Sampling was conducted in July and August 2021 on artificially planted flower strips in the city of Braunschweig, Germany (Table S1; Fig. 1). The sampling period was chosen to target individuals that developed in 2021 at the study sites, as the egg-laying founding female typically remains inside the nest after the emergence of the first brood early in summer [55, 56], making it unlikely that females from the previous year were collected during this period. Braunschweig, located in Lower Saxony, has a population of approximately 250,000 inhabitants (braunschweig.de, 2021) and covers an area of 192 km^2^ (north– south: 19.1 km, west– east: 15.7 km), of which 14% is residential, 8.4% consists of industrial and commercial areas, 7.5% is roads, 36% is agricultural land and 26% consists of green areas (parks, gardens, forests and semi-natural cover) (braunschweig.de, 2020). *Halictus scabiosae* was first recorded in Braunschweig in 2018 [58], most likely due to its expansion northwards [59], and is now common and widely distributed throughout the city.

Using hand-nets, we sampled 158 female *H. scabiosae* individuals from 16 sites (10 individuals per site, one site with 8 individuals). To capture a gradient of urbanisation, these sites were distributed from the city centre to the outskirts (Fig. 1). To ensure independent sampling, sites were spaced at least 1 km apart, a distance exceeding the foraging range of most bee species [33]. Sampling was conducted between 09:00 and 15:00 on days with good weather conditions (sunny, >16 °C, low wind; Table S1). Due to the distinct morphological characteristics of *H. scabiosae*, individuals were identified in the field. Collected bees were kept in 100% ethanol and stored at −20 °C for further analysis.

### Body size measurements

We used the intertegular distance (ITD), the distance between the two wing bases (tegulae) on the thorax, as a proxy for body size [46, 61, 62]. ITD is strongly correlated with the dry body mass of bees [63] and a good predictor of flight ability as it is associated with flight muscle volume [63] and foraging distance [33]. ITD measurements were taken once per individual by J. de V. using a stereo microscope (Olympus SZX7) with an integrated camera, connected to a computer with the cellSens software v.1.6 digital measurement tool. Additionally, we calculated the percentage coefficient of variation (CV = 100 • σ/µ, where σ is the standard deviation and µ is the mean value) of ITD per sampling site.

### Environmental data collection

To determine the main environmental factors influencing body size, we collected various environmental data. To ensure sufficient sample size and consistency, bees were collected from planted flower strips, which served as standardized foraging sites. These strips were established in early 2020, except for one planted in early 2021, all using the same seed mix (Table S2). However, at the time of our sampling, they varied in flower richness and abundance (Table S1). Since body size is primarily influenced by the amount of food available to developing larvae [35], we estimated the availability of floral resources at each sampling site. Although *H. scabiosae* is polylectic, it shows a preference for Asteraceae, Plantaginaceae, Campanulaceae, Convolvulaceae and Dipsacaceae flowers [57, 64]. Therefore, we quantified both flower coverage and species richness, focusing specifically on its main host plants (Tables S2 and S3).

To assess floral resource availability, we placed a 20 m x 20 m quadrat within the flower strip at each site, divided it into four 10 m x 10 m squares. Within each square, we identified the area of 1 m x 1 m with the highest floral resource availability (Fig. S1). For each site, we counted the number of flowering host plant species across all four 1 m x 1 m squares to determine species richness. Flower coverage was assessed by visually estimating the percentage cover of each flowering plant species within each of the four 1 m × 1 m quadrats per site. For each quadrat, the total flower cover was calculated by summing the cover values across all flowering species. The final flower coverage per site was then calculated by averaging the total cover values across the four quadrats. Flowering plants were identified to species level using the Flora incognita app (floraincognita.com), which has a 93% accuracy when multiple images are provided [65]. Floral resource availability was quantified in June, as this period likely influences the development of adult *H. scabiosae* females that were raised in the current year at those sites and subsequently collected in July and August. Across all sites, we identified ten main host flowering plant species, 40% of which were not included in the original seed mix of the flower strips (Table S3). The flower resources measured within the quadrat represent a subset of the total floral availability in the surrounding landscape, but provide a standardised basis for comparing resource availability across sites. Flower species richness and coverage of *H. scabiosae*’s main host plants were used as predictors in subsequent statistical analyses (Tables S2 and S3).

Roads fragment the landscape and may impact bee densities by acting as barriers to foraging and dispersal [66, 67]. A previous study has also found a positive correlation between road density and bee body size [46]. To quantify road density, we measured the total length of roads within multiple spatial radii, increasing in 100 m increments from 100 m to 1,500 m around the centre of each sampling site. Analyses were conducted using Quantum GIS v.3.16.3 (QGIS.org) with road network data obtained from Geofabrik GmbH (download.geofabrik.de).

Land surface temperature (LST) data were obtained using Landsat 8 (landsat.gsfc.nasa.gov/satellites/landsat-8/) using the code provided by Ermida et al. [68]. The Landsat 8 is a thermal infrared sensor with a spatial resolution of 100 m and a 16-day revisit cycle. To capture the main larval development period of *H. scabiosae*, we used median LST data from April to August 2021. LST data were processed using the R packages “terra” [69], “sf” [70] and “exactextractr” [71]. We quantified LST at multiple spatial radii, increasing in 100 m increments from 100 m to 1,000 m around the centre of each sampling site.

We quantified landscape heterogeneity with several metrics that may impact *H. scabiosae* body size and foraging: (i) the proportion of semi-natural cover (e.g. remnant vegetation, shrub land, meadows), (ii) the proportion of managed urban green land uses (e.g. public parks, botanical gardens, allotments, recreation ground like playgrounds and sport fields) and (iii) edge density (i.e. ecotones; transition between two habitat types). These variables were analysed using Quantum GIS with data obtained from Geofabrik GmbH and quantified at multiple spatial radii in 100 m increments from 100 m to 1,500 m.

The effects of landscape on bee body size are likely scale-dependent, as different environmental factors influence body size at varying spatial scales. For instance, local-scale floral resource availability can directly impact larval nutrition and development by determining the immediate foraging success of provisioning females. In contrast, broader landscape features such as semi-natural areas, managed green spaces, road density and edge density, which affect habitat connectivity, as well as nesting opportunities and foraging range operate at larger spatial scales. These broader-scale factors influence overall resource accessibility and movement potentially shaping bee body size through ecological sorting. Since each predictor may exert its influence at a distinct spatial scale, selecting a single scale risks overlooking key relationships. For example, road density may act as a movement barrier at smaller scales (100–500 m) but serve as an indicator of urban fragmentation at larger scales (≥ 1,000 m), potentially filtering for larger-bodied bees that can traverse fragmented landscapes [46]. Similarly, semi-natural areas and managed green spaces may provide critical nesting and foraging habitats at intermediate scales, influencing bee populations differently depending on spatial context.

Studies have shown that a multi-scale approach improves ecological modelling by capturing the most relevant spatial scales for each variable, enhancing our ability to detect patterns and mechanisms driving species responses [72, 73]. By integrating multiple spatial scales, we can better account for the ecological processes influencing *H. scabiosae* body size, ensuring a more comprehensive understanding of how landscape structure affects bee populations. This approach enables us to identify the most predictive scale for each environmental factor, ultimately providing stronger insights into the spatial dynamics of habitat suitability, temperature and fragmentation effects on bee body size.

To identify the spatial scales with the most predictive power for body size shifts in *H. scabiosae*, we correlated our response variables (ITD and CV of ITD) with each of our predictors (road density, LST, proportion of semi-natural cover, proportion of managed green spaces and edge density, Tables S4 and S5). For ITD, the correlation coefficient peaked at the following distances: 1,000 m for road density, 600 m for LST, 900 m for semi-natural cover, 1,500 m for managed green spaces and 1,300 m for edge density. For the CV of ITD, correlation coefficients peaked at 700 m for road density, 600 m for LST, 600 m for semi-natural cover, 1,300 m for managed green spaces, and 1,100 m for edge density. These radii were used in subsequent analyses.

### Statistical analysis

**Fig. 2:**
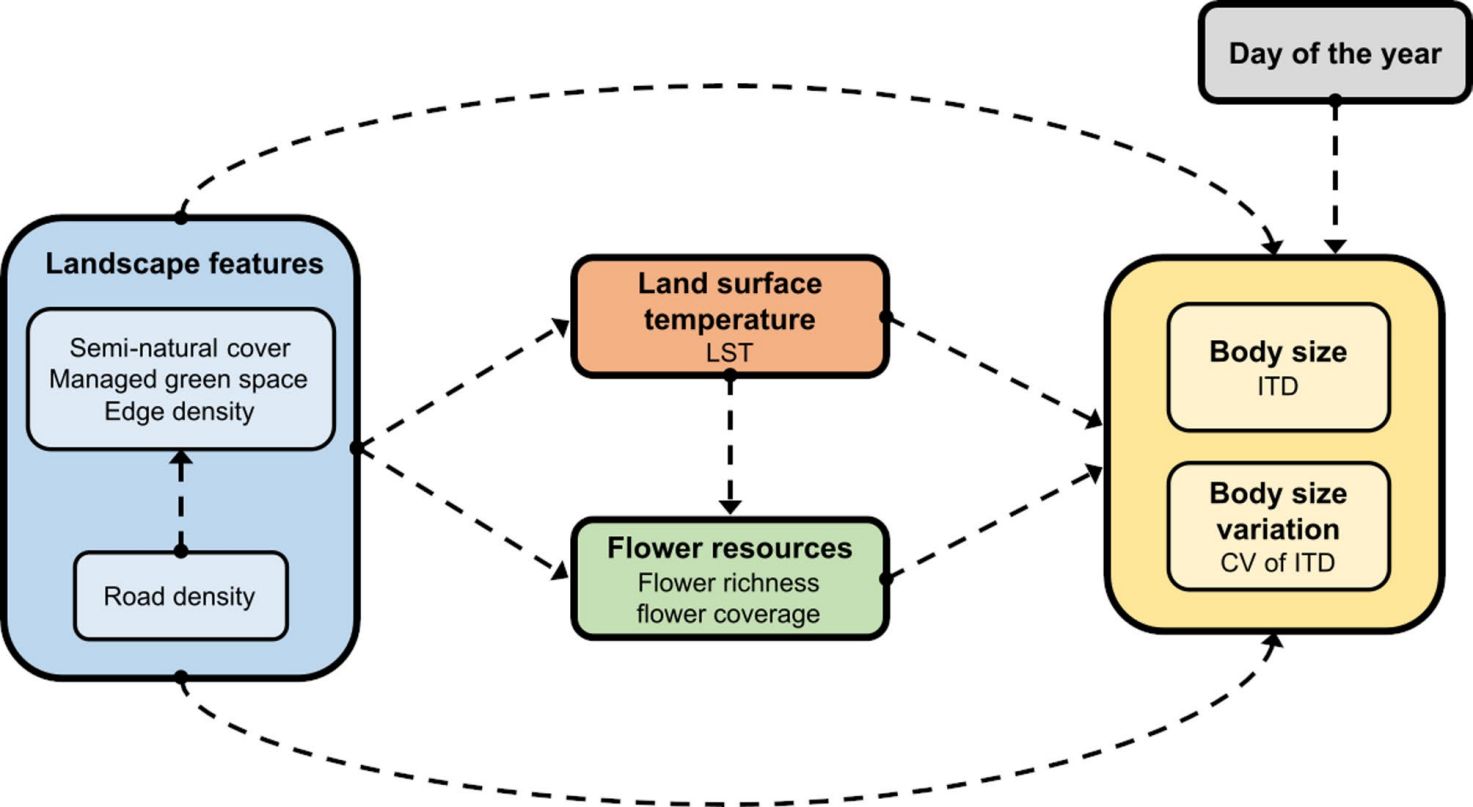
Conceptual directed acyclic graph (DAG) of all hypothesised links among road density and landscape features, temperature (land surface temperature, LST), flower resources, day of the year and body size (ITD in mm) and coefficient of variation of body size (CV of ITD). Road density is hypothesised to affect ITD and CV of ITD either directly or through effects on landscape heterogeneity, temperature and floral resources. Other landscape features (proportion of semi-natural cover, proportion of managed green spaces and edge density) are hypothesised to affect ITD and CV of ITD either directly or through effects on LST and floral resources. LST is hypothesised to affect ITD and CV of ITD either directly or through effects on flower resources. Flower resources and day of the year are hypothesised to affect ITD and CV of ITD directly

All statistical analyses were performed using the R statistical software v.4.3.1 [74]. We used structural equation modelling (SEM), to visualise and statistically test for the importance of predictors and their interrelations in a logical, causal framework. To investigate the effects of road density, LST, proportion of semi-natural cover, proportion of managed green spaces, edge density, main host flower coverage and main host flower species richness on *H. scabiosae* body size, we constructed models based on directed acyclic graphs (DAGs). First, we created a DAG representing our hypotheses (Fig. 2) and then applied piecewise structural equation modelling (piecewise SEM) using the R package “piecewiseSEM” [75]. In piecewise SEM, paths are estimated in individual models and then integrated into a causal model [75].

To build our piecewise SEMs, we first defined our response variables. For our analyses, we used LST, proportion of semi-natural cover, proportion of managed green spaces, edge density, main host flower coverage, main host flower species richness, body size and body size variation as response variables. We then fitted component models to each of the response variables according to their relationships with road density and each other within our full causal path model. We included a potential landscape and LST influence on floral resources, as many of *H. scabiosae*’s host plants were not part of the seed mixes used in the flower strips. For the response variables: landscape, local floral resources, LST and body size variation we used linear models (LMs). For body size we used linear mixed models (LMMs) with site as a random factor. To avoid any potential effects of seasonality and caste on the body size of *H. scabiosae*, we included the day of the year as an additional fixed factor in the models of body size and body size variation. Since we sampled some sites over multiple days, we used the median sampling day of the year for each site.

Once we had created our full model, we used a multi-step model selection process to determine the best, most parsimonious model. We removed weak paths and reassessed the model fit using Fisher’s C test and Shipley’s test of directed separation (d-seperation), both implemented in the R package “piecewiseSEM”. To find the best, most parsimonious model, we dropped predictors and compared the Akaike Information Criterion for small sample sizes (AICc), choosing the model with the lowest AICc. All model assumptions were checked visually with the R package “DHARMa” [76] and were found to conform to expectations (normally distributed residuals, homoscedasticity, linearity, no outliers). Furthermore, we checked for collinearity among the predictors with variance inflation factors (VIFs) with a cut-off of five [77]. No multicollinearity was detected in our models. For the LMMs, we report marginal R², which reflects the variance explained by the fixed effects, and conditional R², which includes both fixed and random effects [78].

## Results

**Fig. 3:**
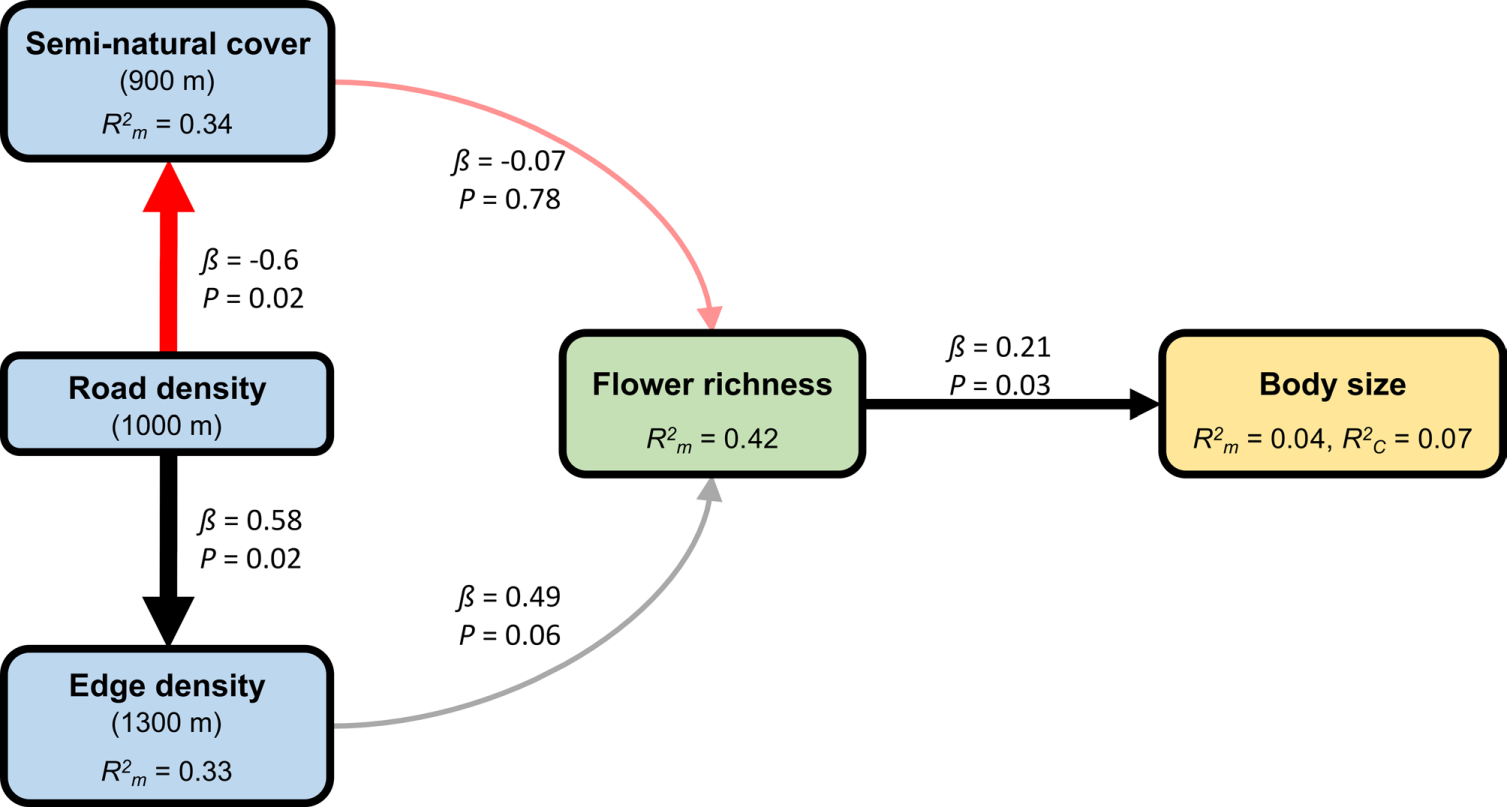
Directed acyclic graph (DAG) of the best piecewise SEM of the hypothesised indirect effect of road density (radius 1,000 m) on body size (ITD, mm) through semi-natural cover (radius 900 m), edge density (radius 1,300 m) and flower richness. The boxes show the measured variables. The red arrows show negative relationships, and the black arrows show positive relationships, as derived from the piecewise SEM analysis. Non-significant (*P* > 0.05) paths are semi-transparent. The thickness of the arrows represents the magnitude of the standardised regression coefficient. Standardised path coefficients and *P*-values are reported next to the arrows, and conditional (with all factors, *R*^*2*^*c*) and marginal (only fixed factors, *R*^*2*^*m*) *R*^*2*^ values are reported for all response variables

**Fig. 4:**
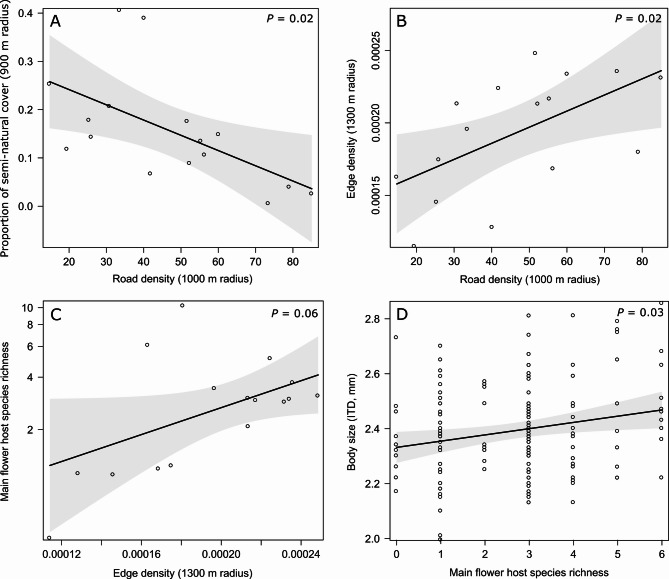
Relationships resulting from the piecewise SEM between **A** the proportion of semi-natural cover at the 900 m radius and road density at the 1,000 m radius, **B** edge density at the 1,300 m radius and road density at the 1,000 m radius, **C** main host flowering plant species richness of *H. scabiosae* and edge density at the 1,000 m radius, **D** *H. scabiosae* body size (ITD, mm) and its main host flowering plant species richness. Plotted lines show predicted relationships. Shaded areas show 95% confidence interval. *P*-values are given in the right, upper corner.

In total, 158 *H. scabiosae* females were measured. The intertegular distance ranged from 1.99 mm to 2.86 mm (x̅ = 2.39 ± 0.17 SD, Table S6) and was normally distributed (Fig. S2). The CV of ITD ranged from 3.3 to 9.4% (x̅ = 6.3 ± 1.6 SD, Table S6). We tested our hypothesis of indirect causal effects of urbanisation on body size with a piecewise SEM. Our best causal model (Fisher`s C = 11.49, *P* = 0.321, df = 10; Table S8) included an effect of road density on edge density, semi-natural cover and flower richness and an effect of flower richness on body size (Fig. 3). Road density had a negative effect on the proportion of semi-natural cover (LM; *β* = −0.587, *P* = 0.019; Fig. 4A, Table S8) and a positive effect on edge density (LM; *β* = 0.576, *P* = 0.019; Fig. 4A, Table S8). In addition, the best model included a non-significant negative effect of the proportion of semi-natural cover (LM; *β* = −0.066, *P* = 0.78; Fig. S3, Table S8) and a positive effect of edge density, though only marginally significant, on *H. scabiosae* host flowering plant species richness (LM; *β* = 0.487, *P* = 0.058; Fig. 4C, Table S8). The main host flowering plant richness had a positive effect on the body size of *H. scabiosae* (LMM; *β* = 0.212, *P* = 0.028; Figs. 4D, Table S8).

**Fig. 5:**
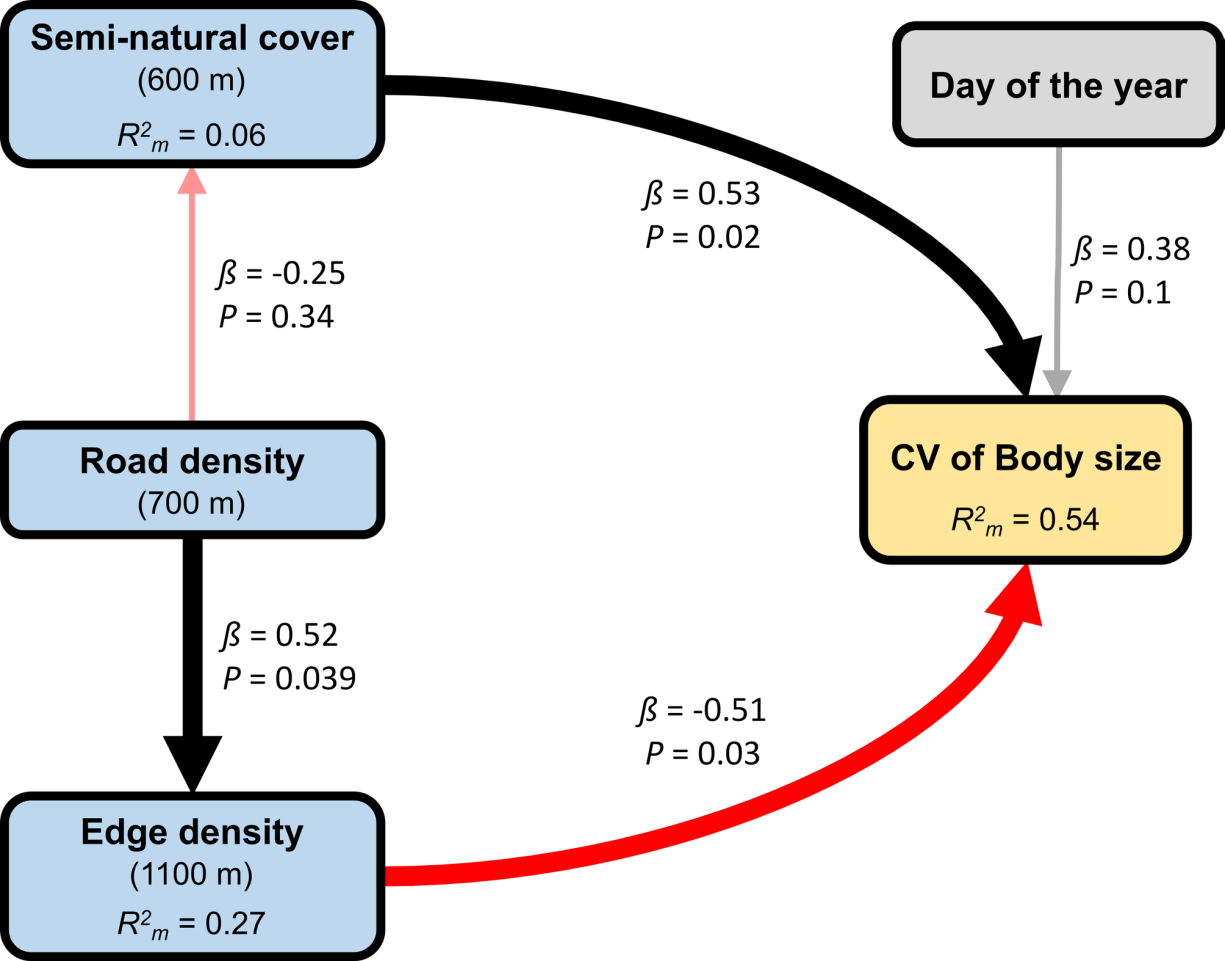
Directed acyclic graph (DAG) of the best piecewise SEM of the hypothesised indirect effects of road density (radius 700 m) on the coefficient of variance (CV) of body size through edge density (radius 1,100 m) and proportion of semi-natural cover (radius 600 m). The boxes show the measured variables. The red arrows show negative relationships, and the black arrows show positive relationships, as derived from the piecewise *SEM* analysis. Non-significant (*P* > 0.05) paths are semi-transparent. The thickness of the arrows represents the magnitude of the standardised regression coefficient. Standardised path coefficients and *P*-values are reported next to the arrows, and marginal *R*^2^ (only fixed factors, *R*^2^_m_) values are reported for all response variables

**Fig. 6:**
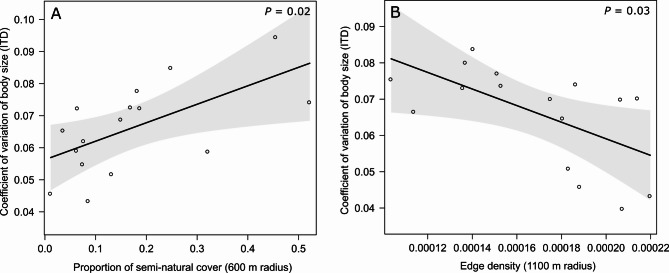
Relationships resulting from the piecewise SEM between **A** the coefficient of variation of body size (CV of ITD) of *H. scabiosae* and the proportion of semi-natural cover at the 600 m radius, **B** the coefficient of variation of body size (CV of ITD) and edge density at the 1,100 m radius. Plotted lines show predicted relationships. Shaded areas show 95% confidence interval. *P*-values are given in the right, upper corner

We tested our hypothesis of indirect causal effects of urbanisation on the coefficient of variance of body size, i.e. CV of ITD, with a piecewise SEM. Our best causal model (Fisher`s C = 5.434, *P* = 0.71, df = 8; Fig. 5, Tabe S9) included an effect of road density on edge density and proportion of semi-natural cover and an effect of edge density, proportion of semi-natural cover and day of the year on the CV of ITD. Road density had a negative effect, although not statistically significant, on the proportion of semi-natural cover (LM; *β* = −0.254, *P* = 0.343; Fig. S4B, Table S9) and a positive effect on edge density (LM; *β* = 0.52, *P* = 0.039; Fig. S4A, Table S9). We found a positive effect of the proportion of semi-natural cover (*β* = 0.529, *P* = 0.022; Fig. 6A, Table S9) and a negative effect of edge density (LM; *β* = −0.511, *P* = 0.031; Fig. 6B, Table S9) on the CV of ITD. The best model further included a non-significant positive effect of day of the year on the CV of ITD (LM; *β* = 0.377, *P* = 0.101; Fig. S4B, Table S9).

## Discussion

In this study, we examined the *H. scabiosae* body size as a response variable within a cityscape. Our findings indicate that host flowering plant richness, the extent of semi-natural cover and edge density are key drivers of *H. scabiosae* body size and its variation. Our best models revealed that *H. scabiosae* body size increases with greater host plant richness. Additionally, body size variation increases with higher semi-natural cover and lower edge density. Below we expand on these results and discuss their ecological and conservation implications.

### Flower richness affects body size

For bees, the quantity and quality of food resources are crucial determinants of body size, as the amount, composition, as well as the nutritional value of larval food directly impact adult size [35, 79]. Several studies have shown that increased pollen quantity and flower abundance lead to larger bee body sizes [40, 80, 81]. However, despite variation in flower abundance among our sample sites, we did not observe a significant effect of flower coverage on *H. scabiosae* body size. This could be because all samples were collected from flower strips where food quantity was likely sufficient.

Food quality is another important component of dietary resources, as low food quality can limit adult body size [79, 82, 83]. Nutritional composition plays a significant role in bee development. For example, the polylectic sweat bee *Lasioglossum zephyrum* produced larger offspring when larvae were fed a protein-rich diet [39]. Similarly, increased protein supply correlated with larger body sizes in the stingless bee *Nannotrigona perilampoides* [40]. Beyond proteins, nutrients like natrium (Na) and zinc (Zn) are also critical for bee body mass [83, 84]. While specific host plants may provide essential nutrients [85], greater flower richness likely increases the probability of balanced larval nutrition. Indeed, *Tetragonula carbonaria* body size has been shown to increase with flower richness [81]. Consistent with these findings, our results show that *H. scabiosae* females exhibited larger body sizes with increasing host plant richness, likely due to enhanced and more diverse larval nutrition i.e. higher food quality.

In our study, the majority of *H. scabiosae*’s primary host plants belonged to the Asteraceae family, which also dominated the floral cover across all sites. Given this consistent dominance, variation in body size is unlikely to result from shifts in plant family composition. Moreover, Asteraceae pollen is generally considered relatively low in protein compared to pollen from other families [86, 87], making it improbable that the body size increase is driven by greater access to high-protein resources. Instead, we propose that greater species richness, even within a single dominant family like Asteraceae, enhances the nutritional diversity available to developing larvae. A more diverse floral assemblage likely provides a broader spectrum of amino acids, micronutrients, lipids, and beneficial secondary metabolites, which together support improved larval development [88] and ultimately lead to larger adult body sizes. Other urban bee studies also support the relationship between food resources and body size. For example, Tommasi et al. [51] found a positive correlation between floral abundance and *Bombus terrestris* body size along an urbanisation gradient. Additionally, our findings align with the hypothesis that shifts in wild bee body size in urban environments, whether increasing [89] or decreasing [48, 49, 90, 91], are primarily driven by floral resource availability.

However, bee body size is also influenced by other environmental factors beyond floral resources. Fragmentation (e.g. road density) and temperature have been shown to affect bee body size [46, 92]. Along an urbanisation gradient, Merckx et al. [93] found a positive correlation between impervious surfaces and micromoth body size, while Tommasi et al. [51] found a negative effect of temperature on *B. pascuorum* body size. However, in our study, neither road density nor temperature significantly influenced *H. scabiosae* body size. This could be due to species-specific responses to urban environmental factors or context-dependent effects of the surveyed city. Notably, *H. scabiosae* is a thermophilic species expanding northward [59, 94, 95] and constructs nests up to 30 cm deep in the ground [53]. Its preference for warm conditions and the insulating effect of subterranean nests may buffer the impact of temperature fluctuations, potentially explaining why surface temperature as determined by us at the landscape level did not affect body size in our study. Future studies should experimentally rear *H. scabiosae* under different food and temperature conditions to better understand their relative influences on body size.

Overall, we did not find a significant direct or indirect effect of urban landscape heterogeneity on body size. While edge density had a marginally significant effect on flowering plant richness, its indirect effect on body size was not significant. Ecotones, characterised by local vegetation, can serve as foraging and nesting habitats for bees [3, 96, 97] and act as pollen transportation corridors, facilitating plant reproduction and connectivity between plant populations [98–100]. In our study, flowering plant richness was not limited to the species included in the seed mix of the flower strips but also included other spontaneously growing species. This suggests that higher edge density may have enhanced landscape connectivity, potentially facilitating seed dispersal and contributing to greater floral richness at these sites.

### Proportion of semi-natural cover and edge density affect variation in body size

Body size variation in a population tends to be higher under stressful environmental conditions [44, 45]. Stressors for wild bees are, for example, increased temperatures due to climate change, exposure to pesticides and pathogens and nutritional stress due to food scarcity and competition [101]. Since adult body size is primarily determined by food during the larval stage, greater size variation may reflect inconsistent food resource availability [35] or competition with other species, e.g. honeybees [102]. In general, increased body size variation may indicate greater environmental instability, shaped by factors like food availability and temperature.

Semi-natural areas are often considered less stressful environments for wild bees because they provide abundant food and nesting resources, supporting higher wild bee diversity and abundance [103–105]. Additionally, they may buffer the negative effects of climate change on wild bee populations [106]. However, we observed that increased semi-natural cover was associated with greater body size variation in *H. scabiosae* females. At a 900 m scale, this effect may be driven by the heterogeneous nature of urban semi-natural areas and the distribution of resources across microhabitats. Variability in vegetation structure and floral resource availability within those semi-natural areas can lead to differences in nutritional intake during larval development, contributing to body size variation. Additionally, while semi-natural areas provide more floral resources, they support higher bee densities and increased species diversity [107, 108], potentially intensifying competition for high-quality or preferred food sources [109]. This competition could lead to uneven access to resources, amplifying differences in body size among individuals. Furthermore, due to increased host density in areas with greater semi-natural cover, bees may experience higher parasite pressures [110, 111], which could impose differential selection on body size [112, 113]. These stressors could alter developmental trajectories and influence parental investment strategies, further shaping body size variation in bee populations. In contrast, higher edge density was associated with decreased body size variation. This could be explained by ecotones promoting plant diversity [98, 99, 114], thereby stabilising food availability and reducing foraging trip duration [115]. Additionally, wild bees frequently use habitat edges as foraging and dispersal routes [116], potentially leading to more stable environmental conditions for *H. scabiosae* in areas of high edge density.

More broadly, it is important to recognize that increased body size variation does not necessarily reflect environmental stress alone. In primitively eusocial species such as *H. scabiosae*, body size variation may also carry adaptive significance by enabling flexible task allocation and division of labour within colonies [35]. In some populations, larger individuals may be better suited to tasks such as long-distance foraging or handling specific floral resources, while smaller individuals may focus more on intranidal tasks such as brood care, nest maintenance, or guarding [35, 117]. This variation can enhance colony resilience, particularly in heterogeneous or unpredictable environments where resource availability and competition fluctuate [35]. However, evidence for size-based division of labour in *H. scabiosae* is mixed; while some populations exhibit clear size-related roles [118], others show no significant size differences between foraging and egg-laying females [53]. This suggests that body size variation in *H. scabiosae* likely reflects a combination of environmental stressors and adaptive developmental plasticity, with the expression of these dynamics varying by ecological context. Importantly, these mechanisms are not mutually exclusive. Furthermore, the threshold at which variation shifts from being adaptive to maladaptive, indicating stress rather than functional flexibility, is context-dependent and remains poorly defined in the current literature [35]. This dual interpretation is particularly relevant to our finding that body size variation increased with greater semi-natural cover, potentially reflecting both the adaptive benefits of task flexibility in heterogeneous environments and the developmental costs associated with uneven or unpredictable resource distribution.

## Conclusion

Wild bees are vital ecosystem service providers with significant ecological and economic value. *Halictus scabiosae* is likely a key pollinator for Asteraceae plants, making its response to urban environments particularly relevant. Body size is one of the most important life-history traits as it is closely linked to fitness, metabolism and dispersal ability [30]. Our findings suggest that, within the urban ecosystem, the availability of diverse or high-quality food resource is a major driver of body size shifts in *H. scabiosae*. To support wild bee populations in urban areas, future conservation efforts should focus on enhancing floral resources and improving habitat connectivity by increasing the amount of ecotones. Specifically, city planners and policymakers should increase and diversify urban floral resources by promoting native plant species, enhance green corridors to provide foraging routes and habitat connectivity. Given that cities can serve as critical refuges for wild bee species, integrating these measures into urban planning will be essential for maintaining pollinator diversity and sustaining ecosystem services in an increasingly urbanised world.

## Supplementary Information

Supplementary Material 1.

## Data Availability

All data are available as supplementary material.
