# Supplementary material for: Local floral resources and edge density within the urban ecosystem promote larger and less variable body size in the great banded furrow bee, Halictus scabiosae
Source: BMC Ecol Evol. 2025 Jul 31;25:75. doi: 10.1186/s12862-025-02416-5 (PMC12312322; doi:10.1186/s12862-025-02416-5)
Supplement: Supplementary file 1 — Supplementary Material 1. [file 12862_2025_2416_MOESM1_ESM.docx]

**Supplementary material**

**Local floral resources and edge density within the urban ecosystem promote larger and less variable body size in the great banded furrow bee, *Halictus scabiosae***

Lucie M. Baltz^1^, Julienne de Vastey^2^, Hanna Gardein^3^, Felix Klaus^3^, Henri Greil^3^, Robert J. Paxton^1,4^, Panagiotis Theodorou^1,4^

^1^ General Zoology, Institute of Biology, Martin Luther University Halle-Wittenberg, Hoher Weg 8, 06120 Halle (Saale), Germany

^2^ Washington & Lee University, 204 W. Washington St., Lexington VA 24450, USA

^3^ Institute for Bee Protection, Julius Kühn Institute (JKI) – Federal Research Centre for Cultivated Plants, Messeweg 11/12, 38104, Braunschweig, Germany

^4^ German Centre for Integrative Biodiversity Research (iDiv) Halle-Jena-Leipzig, Puschstrasse 4, 04103 Leipzig, Germany

^*^ Correspondence:

Lucie M. Baltz, General Zoology, Institute of Biology, Martin Luther University Halle-Wittenberg, Hoher Weg 8, 06120 Halle (Saale), Germany, Email: [baltzlucie@gmail.com](mailto:baltzlucie@gmail.com)

**Supplementary figures**


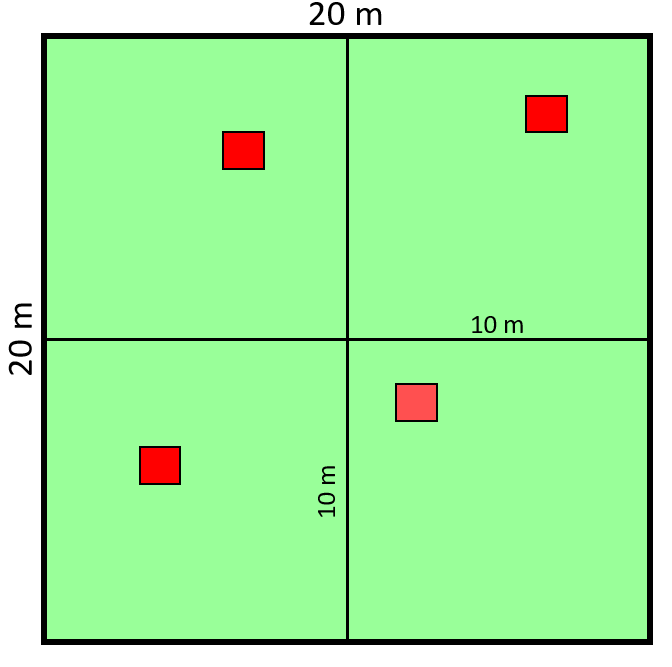


**Supplementary figure S1.** Scheme of the recording method for flowering plant richness and flower abundance. The 20 m x 20 m quadrat was divided into four 10 m x 10 m squares. Within each of these squares, we identified the 1 m x 1 m area with the highest availability of floral resources (red quadrat) and recorded the number of flowering plant species and estimated the percentage of each species flower coverage.


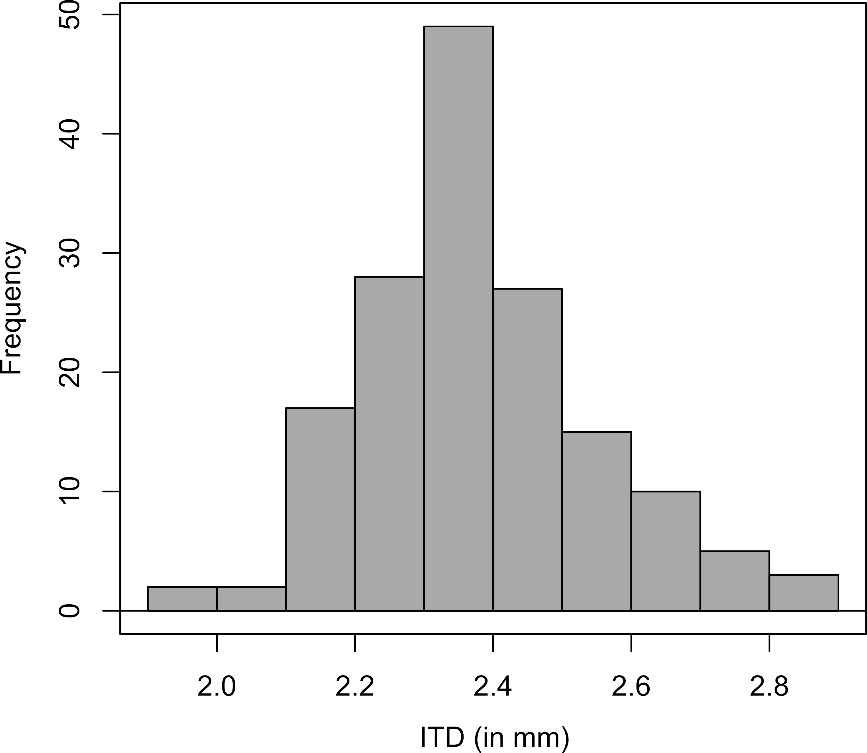


**Supplementary figure S2.** Frequency distribution of the body size of *Halictus scabiosae* (ITD), measured in mm.


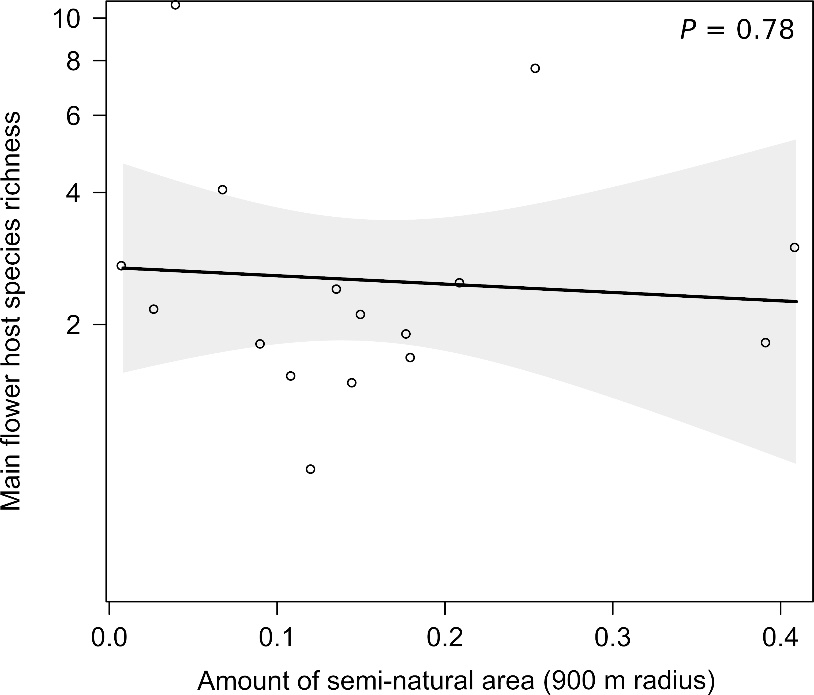


**Supplementary figure S3.** Relationship resulting from the piecewise SEM between the host flowering plant species richness of *Halictus scabiosae* observed in June and proportion of semi-natural area at 900 m radius. Plotted lines show predicted relationships. Shaded area shows 95 % confidence interval. The *P*-value is given in the right, upper corner.


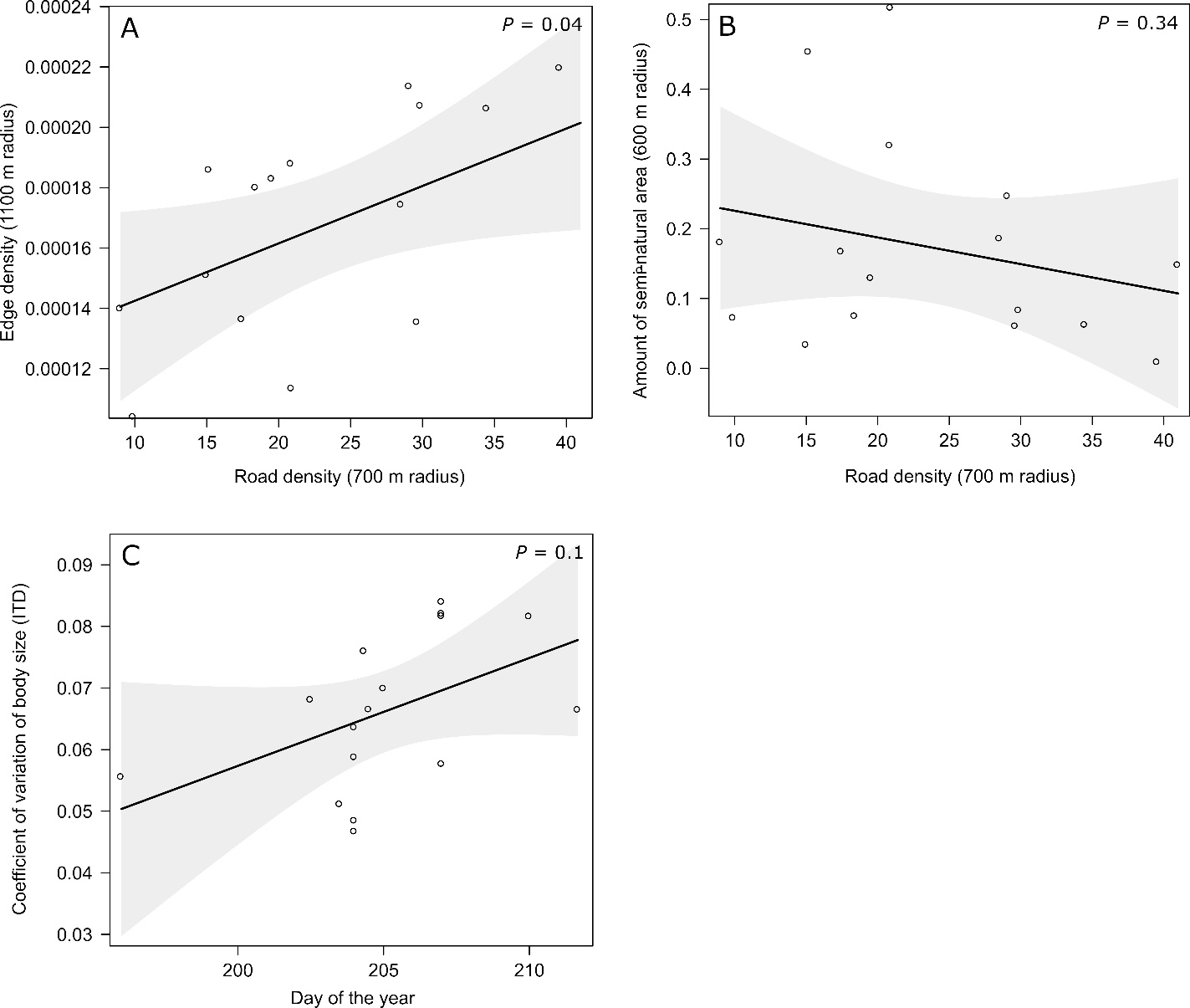


**Supplementary figure S4.** Relationships resulting from the piecewise SEM between A) edge density at the 1,100 m radius and road density at the 700 m radius, B) the proportion of semi-natural area at 600 m radius and road density at 700 m radius and C) coefficient of variation of body size (CV of ITD) and day of the year of sampling. Plotted line shows predicted relationships. Shaded area shows 95 % confidence interval. *P*-values are given in the right, upper corner.

**Supplementary tables**

**Supplementary table S1.** Sites used in our study with coordinates, sampling dates, number of samples and the weather conditions on the sampling day (the average of temperature and wind was used for sites with multiple sampling dates), flower abundance (flower coverage in %) and flowering plant richness of the main host plants of *Halictus scabiosae* in June.

| **Site** | **Latitude (N)** | **Longitude (E)** | **Sampling dates (day/month) in 2021** | **N** | **Weather (at 9 AM)** | | **Flowering plant richness** | **Flower coverage (%)** |
| --- | --- | --- | --- | --- | --- | --- | --- | --- |
|  |  |  |  |  | **Temperature (°C)** | **Wind (km/h)** |  |  |
| AH | 52.34135 | 10.47852 | 20-28/07 | 10 | 17 | 8.5 | 4 | 8.25 |
| AZ | 52.2252 | 10.51585 | 26/07 | 10 | 20 | 6.8 | 5 | 4.25 |
| DO | 52.24382 | 10.48816 | 23/07 | 10 | 16.4 | 2.9 | 1 | 7.75 |
| EM | 52.27504 | 10.50089 | 23-25/07 | 10 | 19 | 4 | 6 | 5 |
| GE | 52.21009 | 10.47436 | 23/07 | 10 | 16.4 | 2.9 | 0 | 0 |
| HB | 52.23748 | 10.53124 | 26/07 | 10 | 20 | 6.8 | 3 | 3 |
| HL | 52.31583 | 10.59968 | 20-27/07 | 10 | 17.8 | 9.9 | 3 | 1.25 |
| JKI | 52.28406 | 10.58228 | 26/07-20/08 | 8 | 18.2 | 8.4 | 3 | 9.5 |
| KK | 52.24538 | 10.5072 | 23/07 | 10 | 16.4 | 2.9 | 3 | 4 |
| LA | 52.26721 | 10.4558 | 29/07 | 10 | 16.5 | 14 | 1 | 0.875 |
| NP | 52.27827 | 10.52751 | 15/07-19/08 | 10 | 18.8 | 10.7 | 4 | 1.625 |
| QU | 52.27973 | 10.55999 | 15/07 | 10 | 19.4 | 8.3 | 3 | 8.375 |
| SA | 52.27948 | 10.46919 | 22-23/07 | 10 | 17.5 | 6.1 | 1 | 0.5 |
| VR | 52.30582 | 10.44191 | 21-22/07 | 10 | 17 | 4 | 1 | 0.125 |
| WM | 52.25744 | 10.52912 | 26/07 | 10 | 20 | 6.8 | 3 | 3.25 |
| WP | 52.25458 | 10.47988 | 23/07 | 10 | 16.4 | 2.9 | 2 | 1 |

**Supplementary table S2.** Seed mix used for the flower stripes, compiled by Henri Greil for the city of Braunschweig. Flower cover that was observed in June is shown as the sum of percentages of flower cover of each quadrat across all sites. Species that were considered main host plant species of *H. scabiosae* [1, 2] are highlighted in bold.

| **Species** | **Flowering period** | **Flower cover** |
| --- | --- | --- |
| ***Achillea millefolium*** | June - October | 27.5 |
| *Anthemis tinctoria* | June - September | 27 |
| *Anthriscus sylvestris* | May - August |  |
| *Barbarea vulgaris* | May - July | 0.5 |
| *Betonica officinalis* | July - August | 1.5 |
| *Campanula rotundifolia* | June - October | - |
| *Campanula trachelium* | July - August | - |
| *Cardamine pratensis* | April - June | - |
| ***Centaurea cyanus*** | June - October | 8.375 |
| *Centaurea jacea* | May - October | - |
| *Centaurea scabiosa* | July - August | - |
| ***Cichorium intybus*** | July - October | 4.25 |
| ***Crepis biennis*** | May - August | 6.875 |
| *Daucus carota* | June - September | 0.5 |
| *Echium vulgare* | May - July | 34.375 |
| *Heracleum sphondylium* | June - September | - |
| *Hypericum perforatum* | July - August | - |
| ***Hypochaeris radicata*** | June - September | 3 |
| *Isatis tinctoria* | May - July | - |
| *Knautia arvensis* | July - August | - |
| *Leontodon autumnalis* | July - September | - |
| *Leucanthemum vulgare* | June - October | 55.625 |
| *Matricaria recutita* | May - August | - |
| *Medicago lupulina* | May - October | 3.625 |
| *Papaver rhoeas* | May - July | 14.75 |
| *Picris hieracioides* | July - October | - |
| *Plantago media* | May - September | - |
| *Ranunculus acris* | May- September | - |
| *Reseda lutea* | May - September | 1.75 |
| *Reseda luteola* | June - September | - |
| *Rhaphanus raphanistrum* | June - October | - |
| *Salvia pratensis* | May - August | - |
| *Scabiosa columbaria* | July - November | - |
| *Sinapis arvensis* | June - October | 1.75 |
| *Tanacetum vulgare* | July - September | - |
| *Teucrium scorodonia* | July - September | - |
| *Trifolium pratense* | June - September | 9.875 |
| *Verbascum lychnitis* | June - August | - |
| *Verbascum nigrum* | June - September | 0.125 |

**Supplementary table S3.** Flowering plant species that were observed in June and not within the seed mix. Flower cover is shown as the sum of percentages of flower cover of each quadrat across all sites. Species that were considered main host plant species of *H. scabiosae* [1, 2] are highlighted in bold.

| **Species** | **Flower cover** |
| --- | --- |
| *Arenaria serpyllifolia* | 0.25 |
| *Bellis perennis* | 1.5 |
| *Berteroa incana* | 0.25 |
| *Capsula bursa-pastoris* | 0.25 |
| *Cerastium fontanum* | 0.125 |
| *Chaenomeles speciosa* | 0.25 |
| ***Cirsium arvense*** | 0.125 |
| *Cota tinctoria* | 14 |
| *Erodium cicutarium* | 0.25 |
| *Geranium molle* | 1.375 |
| *Lactuca serriola* | 0.125 |
| *Leucanthemum* sp. | 12.75 |
| *Lotus corniculatus* | 0.25 |
| *Matricaria chamomilla* | 11.25 |
| *Matricaria* sp. | 2.875 |
| *Myosotis arvensis* | 0.75 |
| *Papaver dubium* | 7.75 |
| ***Plantago lanceolata*** | 0.5 |
| *Potentilla reptans* | 0.25 |
| *Ranunculus repens* | 0.125 |
| ***Senecio inaequidens*** | 1.875 |
| *Stellaria media* | 0.25 |
| ***Taraxacum officinale*** | 6 |
| *Trifolium dubium* | 0.375 |
| *Trifolium hybridum* | 20.875 |
| *Tripleurospermum maritimum* | 1.75 |
| *Trifolium repens* | 2.625 |
| *Veronica arvensis* | 0.125 |
| *Veronica persica* | 0.125 |
| *Vicia sativa* | 0.5 |
| *Vicia villosa* | 0.25 |
| *Viola arvensis* | 0.375 |

**Supplementary table S4.** Correlation coefficients of the different predictors and body size (ITD) and coefficient of variation of body size (CV of ITD). The marked values in bold represent the radii with the highest correlation.

| **Body size** (ITD) | | | | | | | | | | | | | | | |
| --- | --- | --- | --- | --- | --- | --- | --- | --- | --- | --- | --- | --- | --- | --- | --- |
| Radius (in m) | 100 | 200 | 300 | 400 | 500 | 600 | 700 | 800 | 900 | 1000 | 1100 | 1200 | 1300 | 1400 | 1500 |
| Edge density | 0.041 | 0.038 | 0.024 | 0.030 | 0.061 | 0.062 | 0.060 | 0.073 | 0.080 | 0.083 | 0.069 | 0.093 | **0.107** | 0.105 | 0.098 |
| Semi-natural area | -0.031 | -0.120 | -0.120 | -0.116 | 0.038 | 0.083 | -0.144 | -0.151 | **-0.151** | -0.132 | -0.090 | -0.085 | -0.089 | -0.095 | -0.096 |
| Managed green space | 0.126 | 0.090 | 0.072 | 0.063 | 0.082 | 0.093 | 0.098 | 0.107 | 0.119 | 0.126 | 0.134 | 0.138 | 0.140 | 0.153 | **0.156** |
| Road density | -0.008 | 0.014 | -0.005 | -0.003 | 0.011 | 0.024 | 0.021 | 0.041 | 0.059 | **0.063** | 0.056 | 0.055 |  |  |  |
| Surface temperature | -0.039 | -0.025 | -0.030 | -0.039 | -0.040 | **-0.041** | -0.038 | -0.039 | -0.039 | -0.034 |  |  |  |  |  |
| **Coefficient of variation of body size** (CV of ITD) | | | | | | | | | | | | | | | |
| Radius (in m) | 100 | 200 | 300 | 400 | 500 | 600 | 700 | 800 | 900 | 1000 | 1100 | 1200 | 1300 | 1400 | 1500 |
| Edge density | -0.194 | -0.392 | -0.436 | -0.415 | -0.209 | -0.258 | -0.319 | -0.308 | -0.341 | -0.392 | **-0.465** | -0.422 | -0.398 | -0.390 | -0.378 |
| Semi-natural area | 0.058 | 0.111 | 0.219 | 0.239 | 0.458 | **0.521** | 0.190 | 0.146 | 0.117 | 0.123 | 0.066 | 0.048 | 0.013 | -0.045 | -0.088 |
| Managed green space | -0.212 | -0.163 | -0.122 | -0.149 | -0.133 | -0.134 | -0.150 | -0.150 | -0.159 | -0.174 | -0.199 | -0.228 | **-0.246** | -0.231 | -0.219 |
| Road density | -0.418 | -0.412 | -0.398 | -0.413 | -0.417 | -0.405 | **-0.419** | -0.394 | -0.369 | -0.362 | -0.377 | -0.374 |  |  |  |
| Surface temperature | -0.199 | -0.204 | -0.287 | -0.367 | -0.402 | **-0.410** | -0.406 | -0.401 | -0.391 | -0.375 |  |  |  |  |  |

**Supplementary table S5.** The values of the predictors used in the analysis for body size and the coefficient of variation (CV) of body size. In brackets, the radius used for a given predictor is given.

|  | **Predictors for body size** | | | **Predictors for both** | | **Predictors for CV of body size** | | |
| --- | --- | --- | --- | --- | --- | --- | --- | --- |
| Site | Edge density  (900 m) | Proportion of seminatural area (900 m) | Road density (1000 m) in km | Proportion of managed green space (1000 m) | Surface temperature  (600 m) in °C | Edge density (300 m) | Proportion of seminatural area (600 m) | Road density (500 m) in km |
| AH | 0.000125 | 0.255 | 14.8 | 0.016 | 25.4 | 0.000043 | 0.182 | 5.7 |
| AZ | 0.000168 | 0.069 | 41.9 | 0.312 | 25.1 | 0.000044 | 0.455 | 6.8 |
| DO | 0.000106 | 0.109 | 56.3 | 0.093 | 26.6 | 0.000029 | 0.062 | 14.3 |
| EM | 0.000127 | 0.040 | 79.0 | 0.030 | 22.2 | 0.000055 | 0.150 | 19.6 |
| GE | 0.000077 | 0.121 | 19.5 | 0.005 | 25.9 | 0.000051 | 0.074 | 4.9 |
| HB | 0.000176 | 0.178 | 51.7 | 0.181 | 25.7 | 0.000078 | 0.248 | 17.5 |
| HL | 0.000142 | 0.210 | 30.8 | 0.023 | 25.9 | 0.000033 | 0.077 | 10.5 |
| JKI | 0.000156 | 0.409 | 33.6 | 0.046 | 23.9 | 0.000086 | 0.132 | 11.1 |
| KK | 0.000171 | 0.151 | 60.1 | 0.242 | 34.8 | 0.000094 | 0.085 | 17.7 |
| LA | 0.000118 | 0.145 | 26.0 | 0.086 | 23.8 | 0.000034 | 0.036 | 8.5 |
| NP | 0.000189 | 0.008 | 73.4 | 0.133 | 26.0 | 0.000067 | 0.011 | 19.9 |
| QU | 0.000134 | 0.136 | 55.4 | 0.090 | 29.2 | 0.000044 | 0.187 | 13.5 |
| SA | 0.000100 | 0.392 | 40.1 | 0.034 | 21.4 | 0.000049 | 0.522 | 10.9 |
| VR | 0.000121 | 0.180 | 25.4 | 0.008 | 23.9 | 0.000035 | 0.169 | 11.2 |
| WM | 0.000187 | 0.027 | 85.1 | 0.232 | 27.1 | 0.000102 | 0.064 | 17.8 |
| WP | 0.000153 | 0.091 | 52.3 | 0.369 | 17.2 | 0.000057 | 0.322 | 8.7 |

**Supplementary table S6.** The measured intertegular distance (ITD) for each bee individual (ID) for each site.

| **Site** | **ID** | **ITD in mm** |
| --- | --- | --- |
| AH | HS AH-13 | 2.44 |
| AH | HS AH-14 | 2.63 |
| AH | HS AH-15 | 2.26 |
| AH | HS AH-16 | 2.29 |
| AH | HS AH-17 | 2.4 |
| AH | HS AH-18 | 2.59 |
| AH | HS AH-19 | 2.81 |
| AH | HS AH-20 | 2.13 |
| AH | HS AH-21 | 2.59 |
| AH | HS AH-22 | 2.38 |
| AZ | HS-AZ-11 | 2.22 |
| AZ | HS-AZ-12 | 2.39 |
| AZ | HS-AZ-13 | 2.22 |
| AZ | HS-AZ-14 | 2.79 |
| AZ | HS-AZ-15 | 2.49 |
| AZ | HS-AZ-16 | 2.65 |
| AZ | HS-AZ-17 | 2.75 |
| AZ | HS-AZ-18 | 2.26 |
| AZ | HS-AZ-19 | 2.76 |
| AZ | HS-AZ-20 | 2.33 |
| DO | HS DO-11 | 2.16 |
| DO | HS DO-12 | 2.18 |
| DO | HS DO-13 | 2.38 |
| DO | HS DO-14 | 2.37 |
| DO | HS DO-15 | 2.51 |
| DO | HS DO-16 | 2.38 |
| DO | HS DO-17 | 2.53 |
| DO | HS DO-18 | 2.1 |
| DO | HS DO-19 | 2.33 |
| DO | HS DO-20 | 2.49 |
| EM | HS EM-11 | 2.36 |
| EM | HS EM-12 | 2.47 |
| EM | HS EM-13 | 2.4 |
| EM | HS EM-14 | 2.22 |
| EM | HS EM-15 | 2.43 |
| EM | HS EM-16 | 2.86 |
| EM | HS EM-17 | 2.63 |
| EM | HS EM-18 | 2.68 |
| EM | HS EM-19 | 2.51 |
| EM | HS EM-20 | 2.46 |
| GE | HS-GE-11 | 2.3 |
| GE | HS-GE-12 | 2.73 |
| GE | HS-GE-13 | 2.48 |
| GE | HS-GE-14 | 2.26 |
| GE | HS-GE-15 | 2.37 |
| GE | HS-GE-16 | 2.22 |
| GE | HS-GE-17 | 2.34 |
| GE | HS-GE-18 | 2.46 |
| GE | HS-GE-19 | 2.17 |
| GE | HS-GE-20 | 2.32 |
| HB | HS HB-11 | 2.59 |
| HB | HS HB-12 | 2.44 |
| HB | HS HB-13 | 2.63 |
| HB | HS HB-14 | 2.4 |
| HB | HS HB-15 | 2.46 |
| HB | HS HB-16 | 2.81 |
| HB | HS HB-17 | 2.74 |
| HB | HS HB-18 | 2.38 |
| HB | HS HB-19 | 2.19 |
| HB | HS HB-20 | 2.31 |
| HL | HS-HL-11 | 2.67 |
| HL | HS-HL-12 | 2.3 |
| HL | HS-HL-13 | 2.13 |
| HL | HS-HL-14 | 2.37 |
| HL | HS-HL-15 | 2.27 |
| HL | HS-HL-16 | 2.3 |
| HL | HS-HL-17 | 2.33 |
| HL | HS-HL-18 | 2.37 |
| HL | HS-HL-19 | 2.42 |
| HL | HS-HL-20 | 2.39 |
| JKI | HS-JKI-06 | 2.15 |
| JKI | HS-JKI-07 | 2.47 |
| JKI | HS-JKI-08 | 2.35 |
| JKI | HS-JKI-09 | 2.37 |
| JKI | HS-JKI-10 | 2.31 |
| JKI | HS-JKI-18 | 2.13 |
| JKI | HS-JKI-19 | 2.19 |
| JKI | HS-JKI-20 | 2.32 |
| KK | HS KK-11 | 2.36 |
| KK | HS KK-12 | 2.41 |
| KK | HS KK-13 | 2.39 |
| KK | HS KK-14 | 2.33 |
| KK | HS KK-15 | 2.49 |
| KK | HS KK-16 | 2.22 |
| KK | HS KK-17 | 2.3 |
| KK | HS KK-18 | 2.45 |
| KK | HS KK-19 | 2.4 |
| KK | HS KK-20 | 2.33 |
| LA | HS-LA-11 | 2.38 |
| LA | HS-LA-12 | 2.25 |
| LA | HS-LA-13 | 2.56 |
| LA | HS-LA-14 | 2.27 |
| LA | HS-LA-15 | 2.65 |
| LA | HS-LA-16 | 2.51 |
| LA | HS-LA-17 | 2.2 |
| LA | HS-LA-18 | 2.18 |
| LA | HS-LA-19 | 2.39 |
| LA | HS-LA-20 | 2.7 |
| NP | HS NP-11 | 2.27 |
| NP | HS NP-12 | 2.48 |
| NP | HS NP-13 | 2.29 |
| NP | HS NP-14 | 2.37 |
| NP | HS NP-15 | 2.26 |
| NP | HS NP-16 | 2.21 |
| NP | HS NP-17 | 2.22 |
| NP | HS NP-18 | 2.33 |
| NP | HS NP-19 | 2.2 |
| NP | HS NP-20 | 2.49 |
| QU | HS-QU-11 | 2.35 |
| QU | HS-QU-12 | 2.4 |
| QU | HS-QU-13 | 2.53 |
| QU | HS-QU-14 | 2.49 |
| QU | HS-QU-15 | 2.22 |
| QU | HS-QU-16 | 2.25 |
| QU | HS-QU-17 | 2.24 |
| QU | HS-QU-18 | 2.47 |
| QU | HS-QU-19 | 2.17 |
| QU | HS-QU-20 | 2.46 |
| SA | HS-SA-01 | 2.46 |
| SA | HS-SA-02 | 2.61 |
| SA | HS-SA-04 | 2.32 |
| SA | HS-SA-05 | 2.27 |
| SA | HS-SA-06 | 1.99 |
| SA | HS-SA-07 | 2.37 |
| SA | HS-SA-08 | 2.38 |
| SA | HS-SA-09 | 2.42 |
| SA | HS-SA-10 | 1.99 |
| SA | HS-SA-21 | 2.35 |
| VR | HS-VR-11 | 2.01 |
| VR | HS-VR-12 | 2.15 |
| VR | HS-VR-13 | 2.2 |
| VR | HS-VR-14 | 2.24 |
| VR | HS-VR-15 | 2.37 |
| VR | HS-VR-16 | 2.38 |
| VR | HS-VR-17 | 2.59 |
| VR | HS-VR-18 | 2.4 |
| VR | HS-VR-19 | 2.45 |
| VR | HS-VR-20 | 2.49 |
| WM | HS-WM-11 | 2.48 |
| WM | HS-WM-12 | 2.6 |
| WM | HS-WM-13 | 2.63 |
| WM | HS-WM-14 | 2.58 |
| WM | HS-WM-15 | 2.16 |
| WM | HS-WM-16 | 2.35 |
| WM | HS-WM-17 | 2.46 |
| WM | HS-WM-18 | 2.33 |
| WM | HS-WM-19 | 2.39 |
| WM | HS-WM-20 | 2.2 |
| WP | HS WP-11 | 2.33 |
| WP | HS WP-12 | 2.28 |
| WP | HS WP-13 | 2.34 |
| WP | HS WP-14 | 2.56 |
| WP | HS WP-15 | 2.57 |
| WP | HS WP-16 | 2.25 |
| WP | HS WP-17 | 2.32 |
| WP | HS WP-18 | 2.55 |
| WP | HS WP-19 | 2.32 |
| WP | HS WP-20 | 2.49 |

**Supplementary table S7.** The coefficient of variation of body size (CV of ITD) for each site.

| **Site** | **CV of ITD** |
| --- | --- |
| AH | 0.083 |
| AZ | 0.094 |
| DO | 0.065 |
| EM | 0.072 |
| GE | 0.068 |
| HB | 0.078 |
| HL | 0.058 |
| JKI | 0.052 |
| KK | 0.033 |
| LA | 0.078 |
| NP | 0.046 |
| QU | 0.055 |
| SA | 0.084 |
| VR | 0.075 |
| WM | 0.067 |
| WP | 0.053 |

**Supplementary table S8.** Model statistics of the piecewise structural equation model for the coefficient of variation of body size (CV of ITD) of *Halictus scabiosae*.

| **Coefficients** | | | | | | | | | | |
| --- | --- | --- | --- | --- | --- | --- | --- | --- | --- | --- |
| **Response variable** | **Predictor** | ***ß* coefficient** | **Standard error** | **DF** | | **Critical value** | ***P*-value** | | ***ß* standard coefficient** | |
| Seminatural area (900 m) | Road density  (1000 m) | -0.0032 | 0.0012 | 14 | | -2.6578 | **0.0187** | | -0.5970 | |
| Edge density  (1300 m) | Road density  (1000 m) | 1.112E-6 | 4.205E-7 | 14 | | 2.645 | **0.0192** | | 0.5758 | |
| Flower richness | Edge density  (1300 m) | 8886.2385 | 4681.1538 | 13 | | 1.8983 | 0.0577 | | 0.4866 | |
| Flower richness | Seminatural area (900 m) | -0.4397 | 1.5751 | 13 | | -0.2792 | 0.7801 | | -0.0659 | |
| Body size (ITD in mm) | Flower richness | 0.0227 | 0.0092 | 13.13 | | 2.4796 | **0.0275** | | 0.2125 | |
| **Global goodness-of-fit** | | | |  | | | | | | |
| **Fisher’s C** | **P-value** | **DF** | **AICc** |  | |  |  | |  | |
| 11.48 | 0.321 | 10 | -336.968 |  | |  |  | |  | |
| **Individual *R^2^*** | | |  |  | | | | | | |
| **Response variable** | **Marginal *R^2^*** | **Conditional *R^2^*** |  |  |  | | |  | |  |
| Seminatural area (900 m) | 0.34 | NA |  |  |  | | |  | |  |
| Edge density  (1300 m) | 0.33 | NA |  |  |  | | |  | |  |
| Flower richness | 0.42 | NA |  |  |  | | |  | |  |
| Body size (ITD in mm) | 0.04 | 0.07 |  |  |  | | |  | |  |

**Supplementary table S9.** Model statistics of the piecewise structural equation model for the coefficient of variation of body size (CV of ITD) of *Halictus scabiosae*.

| **Coefficients** | | | | | | | |
| --- | --- | --- | --- | --- | --- | --- | --- |
| **Response variable** | **Predictor** | ***ß* coefficient** | **Standard error** | **DF** | **Critical value** | ***P*-value** | ***ß* standard coefficient** |
| CV of ITD | Day of the year | 0.0018 | 0.001 | 12 | 1.7752 | 0.1012 | 0.3766 |
| CV of ITD | Seminatural area (600 m) | 0.0576 | 0.0220 | 12 | 2.6236 | **0.0222** | 0.5292 |
| CV of ITD | Edge density (1100 m) | -228.6910 | 93.4505 | 12 | -2.4472 | **0.0308** | -0.5109 |
| Seminatural area (600 m) | Road density  (700 m) | -0.0038 | 0.0039 | 14 | -0.9827 | 0.3425 | -0.254 |
| Edge density (1100 m) | Road density  (700 m) | 1.903E-6 | 8.36E-07 | 14 | 2.2772 | **0.039** | 0.5199 |
| **Global goodness-of-fit** | | | |  | | | |
| **Fisher’s C** | **P-value** | **DF** | **AICc** |  |  |  |  |
| 5.434 | 0.71 | 8 | -374.296 |  |  |  |  |
| **Individual *R^2^*** | |  |  |  | | | |
| **Response variable** | ***R^2^*** |  |  |  |  |  |  |
| CV of ITD | 0.54 |  |  |  |  |  |  |
| Seminatural area (600 m) | 0.06 |  |  |  |  |  |  |
| Edge density (1100 m) | 0.27 |  |  |  |  |  |  |

**References**

1. Fortunato L, Zandigiacomo P. Fenologia e preferenze florali di *Halictus scabiosae* (Rossi)in Friuli Venezia Giulia. Boll Soc Nat “Silvia Zenari”, Pordenone. 2012; June.

2. Westrich P. Die Wildbienen Deutschlands. 2nd edition. Stuttgart: Verlag Eugen Ulmer; 2019.
